# Therapeutic Potential of Quercetin as an Antiatherosclerotic Agent in Atherosclerotic Cardiovascular Disease: A Review

**DOI:** 10.1155/2020/5926381

**Published:** 2020-06-04

**Authors:** Qian Deng, Xiao Xue Li, Yanting Fang, Xin Chen, Jingui Xue

**Affiliations:** Shuguang Hospital Affiliated to Shanghai University of Traditional Chinese Medicine, Shanghai, China

## Abstract

Atherosclerotic cardiovascular disease (ASCVD) is one of the diseases with the highest morbidity and mortality globally. It causes a huge burden on families and caregivers and high costs for medicine and surgical interventions. Given expensive surgeries and failures of most conventional treatments, medical community tries to find a more cost-effective cure. Thus, attentions have been primarily focused on food or herbs. Quercetin (Qu) extracted from food, a flavonoid component, develops potentials of alternative or complementary medicine in atherosclerosis. Due to the wide range of health benefits, researchers have considered to apply Qu as a natural compound in therapy. This review is aimed to identify the antiatherosclerosis functions of Qu in treating ASCVD such as anti-inflammatory, antioxidant properties, effects on endothelium-dependent vasodilation, and blood lipid-lowering.

## 1. Introduction

Atherosclerosis (AS) is a chronic progressive inflammatory disease associated with inflammatory response, endothelial dysfunction, lipid metabolism disorders, smooth muscle cell migration and proliferation, and oxidative stress [1, 2]. Atherosclerotic cardiovascular disease (ASCVD) caused by AS is one of the diseases with the highest morbidity and mortality in the world, ranking first in the total cause of death among urban and rural residents in China, with more than 40% mortality rate, whose incidence and number of cases are still rising [3]. Although the study shows that in the past 30 years, the incidence of coronary heart disease in the United States has dropped from 6.9% to 5.2%, the mortality rate due to coronary heart disease has dropped 60%, and ASCVD has been and will continue to be the leading cause of death among US residents [4]. Therefore, the treatment of ASCVD is a hot topic concerned by the public. In recent years, a number of research achievements have been made in the field of atherosclerosis [5]. Various medicines, such as HMG-CoA reductase inhibitors (statins) [6], cholesteryl ester transfer protein (CETP) inhibitors (anacetrapib) [7], and cholesterol absorption inhibitors (ezetimibe) [8], have been clinically proven in the treatment of atherosclerosis. Antimyocardial ischemia drugs, coronary angioplasty, coronary stent implantation, and surgical coronary artery bypass grafting have been widely used to treat ASCVD.

Beyond modern medical treatment, the conventional medicine featured by natural herbs with satisfactory clinical efficacy and low toxicity can be used as replacement therapies for many diseases. Relevant research has become a hot topic in modern medicine [9–11]. Natural products like flavonoids which have been proved to have many functions such as anticancer [12], anti-inflammatory [13], antidiabetes [14], antivirus, and antiallergy [15] are considered as important resources in the treatment of cardiovascular disease. It has been widely reported that Quercetin (Qu), as one of the important flavonoids, plays an important role in the prevention and treatment of atherosclerosis. Many biological targets of Qu have been discovered, for instance, inhibiting the formation of reactive oxygen species by blocking nicotinamide-adenine dinucleotide phosphate (NADPH) oxidase [16], preventing the formation of atherosclerotic plaques by upregulating nitric oxide synthetase [17] and stabilizing endothelial atherosclerotic plaque by downregulating matrix metalloproteinase-1 (MMP-1) [18]. Qu serves as one of the important type of flavonoids and is apparently capable of delivering anticipated antiatherosclerotic effects in ASCVD. Research interest for Qu is because of its diverse range of biological properties. For this review, we have assessed the literature which have been published in PubMed and the Web of Science related to AS or ASCVD in recent years to uncover the protective roles of Qu in antiatherosclerosis and ASCVD. The combined functions of Qu allow for the prevention of AS. This review describes the possible therapeutic benefits of Qu, along with its potential mechanisms of action, to support the clinical use of the Qu for the prevention of ASCVD via antiatherosclerosis.

## 2. Pharmacology and Bioavailability of Quercetin

**Figure 1:**
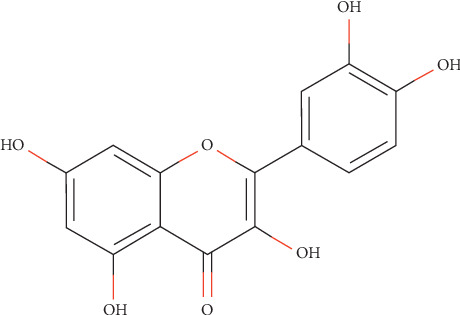
Compound structure of quercetin. Source: http://www.swisstargetprediction.ch.

**Figure 2:**
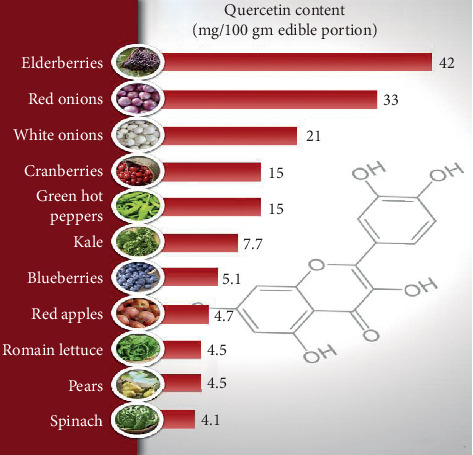
Quercetin content in different foods. Source: http://drjockers.com.

Quercetin (3,3′,4′,5,7 pentahydroxy flavone) is one of a group of over 4000 naturally available plant phenolic compounds whose isolation and biological recognition were first described by Rusznyak and Szent-Györgyi in 1936 [19]. Its chemical structure is an unconjugated aglycone that does not have a carbohydrate moiety and consists by a fused ring system with a benzopyran associated with an aromatic ring and phenyl substituents (the chemical structure is shown in Figure 1). Qu is the most common and widely distributed flavonol compound in our regular diet. This situation is shown in Figure 2. It can be found in almost all plant food, such as tea, onion, lettuce, broccoli, beans, fruits, and buckwheat, and it is also one of the effective components of gingko leaves, mulberry parasitic, sandalwood, and other Chinese herbs [20–22]. The natural flavonol exists in a glycosylated form generally with glucose as its corresponding sugar part. Glycosylation may occur at any of the five OH groups of the flavonol ring additional types of Qu are usually Qu aglycone, such as Qu 3-O-glucuronide and Qu 3′-O-sulfate [23], all kinds of Qu are consumed in the small intestine and digestive tract, the most widely recognized Qu glycoside exhibits the sugar moiety and structures speak to 60–75% of flavonoid intake [24]. With the development of research, modern research has confirmed that Qu has exhibited high antioxidative, anti-inflammatory, and antimicrobial activities [25]. Besides, recent studies have found that Qu can restrain the proliferation and metastasis of multiple cancer cell types, such as breast cancer [26], colon cancer [27], lung cancer [28], and pancreatic cancer cells [29].

Qu has a long history of cardioprotective ability, and it has been extended for clinical trials. In addition to its well-known heart-protecting effects, Qu has also been reported to exert various other pharmacological activities such as anxiolytic [30], hypoglycemic [31], immunomodulatory [32], neuromodulation [33], and wound healing [34]. However, poor water solubility and low bioavailability of Qu have been its limitations. Qu is quickly conjugated with glucuronic acid and/or sulfate during first-pass metabolism (intestine-liver), and a portion of the metabolites is also methylated; the first investigation on the pharmacokinetics of Qu in humans suggested very poor oral bioavailability after a single oral dose (∼2%) [35]. The estimated absorption of Qu glucoside, the naturally occurring form of Qu, ranges from 3% to 17% in healthy individuals receiving 100 mg [36]. Some modified dosage forms of Qu have been developed to overcome these disadvantages, mainly including nanoparticles [37], microemulsions [38], and other drug carriers [39].

## 3. Role of Quercetin in Antiatherosclerosis

### 3.1. Protective Role in Endothelium Protection

Endothelial cells are a semipermeable barrier between blood and vascular wall; it can locally regulate the blood vessels by secreting vasodilator substances such as nitric oxide (NO), prostacyclin, endothelium-derived hyperpolarizing factor, and vasoconstrictive substances such as endothelin, an endothelium-derived contractile factor [40]. These substances can dilate blood vessels, antiproliferate, antiagglutinate, and limit blood pressure in physiological situation, while they can destroy the endothelial homeostasis in pathological states, such as local elevated tensile stress, hypercholesterolemia, total triglyceride- (TG-) rich residual lipoproteins, circulation of vasoactive amines, and infection and immune complex [41]. Qu can relieve vascular endothelial injury through multiple approaches to play a part in antiatherosclerosis [42–44].

NO is an important endogenous vasodilator produced by endothelial nitric synthase (eNOS) acting on L-arginine, which can regulate vascular dilatation and endothelial function [40]. However, when the vascular endothelial cells are affected by a series of harmful factors, the release of vasomotor factors by the endothelial cells decreases, while vasoconstrictors increase, which breaks the vascular homeostasis and causes endothelial dysfunction and eventually leads to the occurrence of AS events [40]. Excessive NO can upregulate the expression of Bcl-2/Bax of mitochondrial through the intracellular Akt pathway, which inhibit the release of reactive oxygen species (ROS), cytochrome c, capase-9, and caspase-3. Qu can reverse the endothelial damage caused by excessive NO by inhibiting nitrification stress and protect endothelial cells [45]. Early studies have found that ATP can increase the shear stress generated by blood flow on vascular endothelial cells, increase the intracellular calcium concentration and the activity of eNOS, thus raise the production and release of NO, and ultimately lead to AS [46]. Qu can inhibit the promoting effect of ATP on NO production in vascular endothelial cells, reduce intracellular calcium concentration and eNOS activity, and reduce vascular endothelial injury [42]. Impaired vascular homeostasis caused by decreased bioactivity of endogenous NO is the most typical pathophysiological feature of endothelial dysfunction [41]. Stabilizing intravascular homeostasis may be an important mechanism, for Qu plays a part in protecting vascular endothelial function.

Oxidative stress is the main cause of endothelial dysfunction, which can lead to an imbalance between endothelial cell membrane stability and permeability, impair the function of endocrine and paracrine, and increase the expression of adhesion molecules and the release of endogenous reactive oxygen species (such as superoxide anion and hydrogen peroxide) [47]. In vitro studies have shown that Qu can directly act as an antioxidant, effectively scavenge oxygen free radicals, and protect vascular endothelial function [48]. Oxidative stress induced by homocysteine (HCY) can lead to endothelial damage [49]. Qu can protect the vascular endothelium from oxidative stress induced by homocysteine by inhibiting lipid peroxidation, protein oxidation, and enzymatic reaction, reducing the level of malondialdehyde (MDA) and increasing the content of glutathione (GSH) in high HCY rats [50].

### 3.2. Lipid Metabolism-Modulating Properties

Dyslipidemia is the main risk factor of AS, including the increase of lipid level caused by total cholesterol (TC), TG, and LDL and the reduction of atherosclerotic lipid levels caused by HDL. Regulating the disorder of lipid metabolism and reducing lipid accumulation are important means to prevent and alleviate AS. In recent years, studies at home and abroad have found that Qu can regulate the metabolism of lipid substances. Some studies have shown that monosodium glutamate (MSG) induces oxidative stress and hepatotoxicity in rats [51] as well as changes the lipid profile of mice [52], and Qu can maintain normal cholesterol levels and prevent the formation of atherosclerotic plaques by treating the dyslipidemia caused by MSG [53]. Besides, Qu can regulate lipid metabolism by lowering the level of TC, TG, LDL, and VLDL in serum and tissues and increasing the content of HDL in serum and phospholipid in tissue [54]. The possible mechanisms of Qu regulating lipid metabolism is described as follows.

HMG-CoA (5-hydroxy-3-methylglutaryl-coenzyme A) reductase is the rate-limiting enzyme in cholesterol synthesis, and its catalytic substrate HMG-COA generating mevalonate is the rate-limiting step in the synthesis of cholesterol. HMG-CoA reductase activity can affect the cholesterol synthesis. It has been demonstrated that Qu can prevent the accumulation of lipid and glycoprotein components in myocardial infarcted rats by reducing the levels of cholesterol, triglycerides, and free fatty acids and decreasing the activity of plasma and liver HMG-CoA reductase [55]. Leptin is a protein hormone involved in the regulation of energy intake and expenditure by the body. Leptin is an important cytokine that plays a key role in regulating lipid metabolism, promoting inflammation, and accelerating aortic valve calcification in patients with coronary heart disease [56]. Mzhelskaya et al. found that Qu can reduce cholesterol levels and lipid levels in high fat and fructose diet rats by regulating leptin-mediated lipid reception [57]. But, the study does not directly clarify the attenuating effect of Qu on cholesterol synthesis; the possible mechanism is that Qu regulate the negative feedback mechanism between fat deposits and fat catabolism rate through leptin, thus promoting the process of lipid metabolism and glucose metabolism and reducing cholesterol intake in rats [57].

Reverse cholesterol transport (RCT) is a process of transporting cholesterol to the liver for further excretion, which can effectively remove excess cholesterol in cells. RCT of macrophages in atherosclerotic plaques is a critical mechanism of antiatherosclerosis. Liver X receptor *α* (LXR*α*) [58], scavenger receptor B-I (SR-BI) [59], and ATP-binding transporter A1 (ABCA1) play a vital role in promoting macrophages RCT and maintaining intracellular cholesterol homeostasis. Many compounds and extracts of traditional Chinese medicine can act on different targets of RCT and exert antiatherosclerosis effect [60]. Qu could promote intracellular cholesterol efflux, upregulate the expression of SR-BI, and reduce the ability of high-density lipoprotein (HDL) to selectively acquire cholesterol in a dose and time-dependent manner, indicating its protective role in atherosclerosis [61]. Peroxisome proliferator-activated receptor *γ* (PPAR *γ*) and LXR*α* are important transcriptional factors for ABCA1. Qu-induced upregulation of ABCA1 and cholesterol efflux of macrophages may be mediated by increased expression levels of the PPAR *γ* and LXR*α* genes [62, 63]. It was shown that Qu can induce the expression of ABCA1 in THP-1 derived foam cells, enhance the apoA-I-dependent cholesterol efflux, and reduce the risk of atherosclerosis [63]. The selective uptake of HDL-C into hepatocytes and steroidogenic cells is the last step of RCT, a process that promotes HDL-C clearance in the plasma. Low clearance of plasma HDL-C aggravates the development of AS while high clearance of plasma HDL-C impedes atherogenesis. A study found that Qu promotes the selective uptake of HDL-C by enhancing SR-BI expression through stimulating the PPAR *γ*/LXR*α* pathway in HepG2 cells [61]. Besides, Qu can also affect the expression of LXR*α* and ABCA1 through the p38-dependent pathway [64], proprotein convertase subtilisin/kexin type 9 (PCSK9) pathway [65], and cholesterol 7 alpha-hydroxylase (CYP7A1) pathway [66] to promote cholesterol efflux and reduce the risk of atherosclerosis.

Accumulation of foam cells and formation of lipid streaks are the early lesion characteristics of AS. Oxidized low-density lipoprotein (oxLDL) is thought to transform native lipoprotein into an antigenic factor that attracts monocyte-derived macrophages to the vascular wall, thereby initiating a convoluted immune response governed by inflammatory modulators [67]. Inhibiting low-density lipoprotein (LDL) oxidation of macrophages and foam cell formation are effective strategies of antiatherosclerosis. Mulberry leaf rich Qu inhibited the oxidation and lipid peroxidation of LDL and attenuated atherosclerosis [68]. An earlier study reported that Qu also targeted the oxLDL-independent pathway and lipopolysaccharide-dependent pathway of lipid droplet formation in macrophages and inhibited the reactive oxygen species production and interleukin (IL)-6 secretion, which indicated the antiatherosclerotic activity of Qu [69]. oxLDL can induce excessive production of reactive oxygen species and block the expression of lectin-like oxidized LDL receptor-1 (LOX-1) in cultured macrophages, leading to lipid accumulation. It was demonstrated in cultured macrophages that Qu could ameliorate lipid deposition and overproduction of reactive oxygen species induced by oxLDL and block the expression of LOX-1 [18].

### 3.3. Anti-Inflammatory Activity

AS is a chronic inflammatory disease mediated by a network of proinflammatory cytokines. Macrophages are critical in the inflammatory response. OxLDL-activated macrophages can synthesize and secrete many growth factors and pro-inflammatory factors, such as platelet-derived growth factor, fibroblast growth factor, and tumor necrosis factor (TNF)-*α*, IL-1, which can promote the growth of plaque and inflammatory response [70, 71]. T cells entering the intima are activated by recognizing antigens presented by macrophages and dendritic cells, producing factors with strong atherogenic effects [2], such as gamma interferon, TNF, and lymphotoxin. There are multiple signaling pathways for Qu to inhibit the expression of inflammatory factors to stabilize atherosclerotic plaque, slow down the progression of atherosclerosis, and reduce the incidence of ASCVD.

A major signaling mechanism associated with atherosclerosis is the activation of NF-*κ*B family of transcription factors. NF-*κ*B is an oxidative stress-sensitive transcription factor that has been found in the early lesions of atherosclerosis [72]. The transcription of vascular cell adhesion molecule-1 (VCAM-1), intercellular adhesion molecule-1 (ICAM-1), and other adhesion molecules, such as E-selectin and P-selectin, is regulated and activated by NF-*κ*B [73]. These activated adhesion molecules help blood-borne cells to recruit to atherosclerotic lesions. Oral administration of Qu significantly suppressed the levels of IL-1*β*, TNF-*α*, and IL-10 in serum and attenuated atherosclerotic plaque through decreasing the transcriptional activity of the NF-*κ*B in patients with coronary artery disease [74]. Qu also inhibited proatherogenic mediators such as ICAM-1 and VCAM-1 in oxLDL-induced smooth muscle cells [32] and downregulated expression of adhesion molecules and NF-*κ*B in TNF-*α*-induced human aortic endothelial cells [75]. Another study demonstrated that Qu is effective to regulate the atherosclerotic inflammatory process by attenuating the NF-*κ*B signaling pathway in endothelial cells and decrease the inflammatory process induced by hypercholesterolemic diet in atherosclerotic rats [32].

Phosphatidylinositol 3-kinase (PI3K) signaling is a multicell surface receptor that regulates cell proliferation, survival, and death. Akt activated by PI3K in response to growth factors or cytokines is an inactive cytoplasmic protein that is recruited to the plasma membrane. The activation of the PI3K/Akt pathway significantly promotes macrophage polarization, which stimulates or inhibits inflammatory responses [44]. Toll-like receptors (TLR) and ROS can mediate the activation of PI3K/Akt signaling pathway [76]. Qu significantly downregulated the elevated mRNA expression of TLRs and cytokine TNF-*α* in HCD-fed atherosclerotic rats in vivo [77]. As Qu possesses inhibition on both TLR-NF-*κ*B signaling pathway and TLR-mediated MAPK pathway, it is evident that it could be used as a therapeutic agent to ameliorate atherosclerotic inflammation and improve ASCVD patients' symptoms [77]. Qu also displayed an inhibitory role in vitro in LPS-induced ROS production, inflammatory response, and apoptosis, which were linked with PI3K/AKT-regulated caspase-3 and NF-*κ*B activation and reduced the atherosclerotic plaque size in high fructose feeding-induced mice [78].

Activation of p38 can activate the expression of cytokines involved in proinflammatory signaling and local recruitment of immune cells, such as E-selectin [79], VCAM-1 [79], and chemokine monocyte chemoattractant protein 1 (MCP-1) [80]. Frei Balz's team found that Qu play an anti-inflammatory role in inhibiting the activation of p38, reducing the expression of E-selectin and ICAM-1 protein in human aortic endothelial cells induced by lipopolysaccharide [81]. Qu also delayed the progression of atherosclerosis by increasing phosphorylation of p38 by activating transforming growth factor beta kinase 1 and mitogenic kinase 3/6 [64].

### 3.4. Antiapoptosis Activity

Apoptosis is an important factor during atherosclerotic development. Autophagy protects vascular cells in plaques against oxidative stress and apoptosis by degrading damaged organelles, which is an effective response against inflammation and oxidative stress in AS plaque cells, and thus it plays an important role in the initiation and development of AS [82]. TNF-*α*, IL-1*β*, and IL-18 are the key inflammatory factors between autophagy and inflammation. A previous study revealed that Qu stabilizes AS plaques and inhibits AS development by reducing the production of the proinflammatory cytokines TNF-*α*, IL-1*β*, and IL-18, a mechanism which involves the induction of autophagy [83]. Weakened autophagy takes place in all major cell types in AS plaques, resulting in impaired macrophage apoptosis and increased senescence of blood vessel endothelial and smooth muscle cells. mTOR, a key molecule in the process of autophagy induction, integrates multiple upstream signaling pathways and interacts with regulator proteins [84]. It also reported that Qu could downregulate the expression levels of mTOR, P53, and cyclin-dependent kinase inhibitor 1A (P21), enhance autophagy, and delay senescence [83]. Autophagy has also been termed as lipophagy, which involves the degradation of intracellular lipid droplets and contributes to cellular lipid metabolism [85]. Another study found that Qu effectively reduces oxLDL-induced RAW264.7 foam cell formation, reducing cellular lipid accumulation and delaying cell senescence through the MammalianSte20-like kinase1 (MST1) pathway [86]. MST1, a novel regulator of apoptosis, participates in multiple biological activities of cells, including autophagy, apoptosis, and oxidative stress [87, 88].

### 3.5. Alteration of the Gut Microbiota and Reduction of Atherogenic Lipid Metabolites

Recent studies of pathogenesis have highlighted the significant roles of the intestinal microbiota and chronic inflammation in both the onset and development of AS [89–91]. The occurrence of AS is strongly associated with inflammatory response caused by the disorder of the intestinal immune system [92]. AS patients have metabolic characteristics, for the community composition of their intestinal flora is different [93]. Inhibiting the inflammatory response and regulating the lipid metabolism of intestinal microorganisms can be used as strategies to delay the development of AS. Short-chain fatty acids (SCFAs) are metabolites of systemic inflammation and are associated with the expansion and rupture of AS plaques [94]; Qu can increase the concentration of SCFAs in the intestinal tract and inhibit the progression of AS [95]. A microbiome with high abundances of Firmicutes and Actinobacteria can result in atherosclerotic area [96, 97]; oral administration Qu reduced the levels of atherogenic lipid metabolites by modulating the abundances of Firmicutes and Actinobacteria [98].

### 3.6. Elimination of Etiology, Reduction of Complications, and Effective Prevention and Treatment of AS

In hemodynamically activated arterial regions, free radical-induced lipoprotein oxidation, enzymatic changes in the intima, production of active lipids, and increased enzymatic activity of MDA and ROS can increase the inflammatory response of arterial intima, thus causing atherosclerotic lesions [99]. Obesity and hypercholesterolemia increase the production of oxygen free radicals and cause oxidative stress [100]; Parvin et al. [101] found that Qu can reduce the blood lipid in obese rats, reduce the expression of oxidative stress factors, improve the vascular remodeling, and prevent the formation of atherosclerotic plaque through antioxidant activity.

In addition to the activities mentioned above, Qu also exerts antithrombotic activity both in vitro and in vivo [102, 103]. Isorhamnetin and tamarixetin inhibit platelet function and thrombus formation through effects on early activation processes including calcium mobilization, granule secretion, and integrin activation [104]. According to the recommendations of the ASCVD Global Guidelines, antiplatelet and antithrombotic treatment is a critical approach for the treatment of acute coronary artery syndrome and secondary prevention of coronary heart disease [105, 106]. Aspirin is one of the most widely used antiplatelet agents worldwide. Its use is associated with adverse events such as gastric bleeding, and whilst studies showed that decreasing aspirin doses reduces adverse event numbers without a reduction in antiplatelet efficacy, even low-dose aspirin treatment is associated with an increased bleeding risk [107]. As such, the pharmacological implications of the effects of the methylated metabolites of Qu with respect to the antiplatelet effects of aspirin are worthy of consideration. Apart from these actions, one more recent study showed that Qu processes an antiapoptotic effect in ischemic myocardial damage [108]. Altogether, these studies demonstrated that Qu may be a promising drug for the treatment and prevention of atherosclerosis in ASCVD.

**Figure 3:**
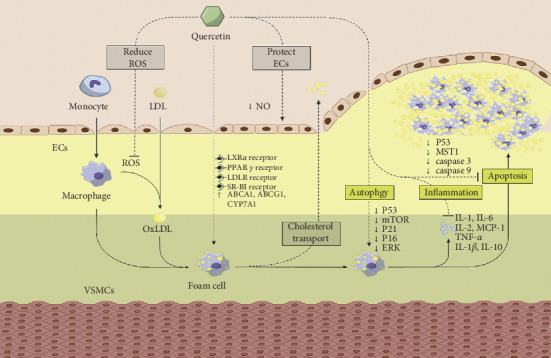
The possible pathway of quercetin on anti-atherosclerotic function.

Taken as in vitro and ex vitro together, the possible pathway of Qu on anti-atherosclerotic function is shown in Figure 3.

Qu can reduce the release of ROS and NOS to protect endothelial cells, improve the expression of ABCA1, ABCG1, and CYP7A1, promote cholesterol efflux in macrophages, downregulate the expressions of P53, P21, P16, and ERK, enhance autophagy to antiapoptosis, and inhibit MCP-1 as well as inflammatory cytokines including IL-1, IL-2, IL-1*β*, IL-6, and TNF-*α* (Tables 1 and 2).

LPO, lipid peroxidation; ABCG1, ATP-binding cassette subfamily G member 1; LPC 18 : 1, lysophosphatidylcholine; IL-6, interleukin 6; SREBP-1c, sterol regulatory element binding protein-1c; HMGR, HMG-CoA reductase; ERK1/2, extracellular signal-regulated kinase (ERK) 1/2; ASAT/ALAT, aspartate amino transferase/alanine amino transferase; CD36, cluster of differentiation 36; GSH, glutathione; CAT, catalase; Dab2, disabled 2; Ldlr^−/−^ mice, low-density lipoprotein receptor null mice; PON1, paraoxonase 1; CVD, coronary artery disease.

COX-2, cyclooxygenase-2; SOCS3, suppressor of cytokine signaling 3; STAT3, signal transducers and activators of transcription 3; Src, steroid receptor coactivator; VSMCs, vascular smooth muscle cells; DCs, dendritic cell; MPMs, mouse peritoneal macrophages, NRK, Normal rat kidney; EPCs, Endothelial progenitor cells; HUVECs, human umbilical vascular smooth muscle cells; PBMCs, Peripheral blood mononuclear cells; HaVSMCs, human aortic smooth muscle cells; EA.hy926 cells, an immortalized HUVEC line derived from fusion of HUVECs and lung adenocarcinoma cells; THP-1, human acute monocytic leukemia cell line.

## 4. Conclusion

In the treatment of ASCVD, Qu can reduce the formation of AS plaque through anti-inflammatory, antioxidation, regulation of lipid metabolism disorders, and other pharmacological effects, therefore reducing the incidence of ASCVD and improving the prognosis of ASCVD patients. In spite of medicine, surgery and intervention operation can improve the cardiac function; it still causes a considerable burden on families and caregivers and results in huge financial costs. Moreover, the high cost of surgery treatments and the failure of most conventional treatments have led the medical community to pursue cost-effective prevention and treatment. For ASCVD patients who are intolerant to surgery and cannot afford the high economic cost of treatment, it is a good choice to increase the Qu-rich low-fat diet in diet. Qu is rich in sources and has a wide range of pharmacological effects such as antitumor, antivirus, antiallergy, and antibacterial effects, which possess a broad prospect in application.

Although the new formulation of Qu has been developed and is partially used in clinics, due to its low bioavailability and low absorption rate, there are still many problems to be solved. Researchers need to further explore the pharmacological mechanism of Qu in human body so as to apply it to the prevention and treatment of clinical diseases better. It is also important to increase the water solubility and oral bioavailability of Qu in the future research. In the further study of phytochemistry, the research field of medicinal activity of Qu should be highlighted for its multiple biological characteristics.

## Figures and Tables

**Table 1:** Summary of the main effects of quercetin on antiatherosclerosis in vivo.

Effect	Subjects	Possible mechanism	Reference
*Animals*			
Endothelial protection	Wistar rats	Neutralize ROS, protect mitochondrial membrane, reduce LPO, and increase GSH levels through the increased catalase activity	[109]
	ApoE^−/−^ mice	Regulate NADPH oxidase subunits expression	[16]
	Sprague-Dawley rats	Increase GSH, erythrocyte CAT, and MDA levels	[50]
	Wistar rats	Decrease serum LDL, TC, MDA, and ROS to improve the vascular structure and prevent from the plaque formation	[101]

Anti-inflammation	ApoE^−/−^ mice	Decrease serum TNF-*α* and IL-6 levels and increase levels	[110]
	ApoE^−/−^ mice	Inhibit phenotypic and functional maturation of DCs	[111]
	Balb/c mice	Mediate HDL function and lipid-glucose state in the circulation by improving biological activity (quality) and the activity of bound to PON1	[112]

Antiapoptosis	Nude mice	Activate ERK and the ERK signaling pathway to promote autophagy	[113]
	ApoE^−/−^ mice	Enhance autophagy and downregulate mTOR, P53 and P21 protein expression levels to alleviate AS lesions and reduce lipid accumulation and increase ratio of LC3 II/I	[83]

Regulate lipid metabolism and reduce the atherosclerotic plaque area	ApoE^−/−^ mice	Increase ABCA1 and LXR*α* expression and downregulate PCSK9 protein expression	[110]
	Wistar rats	Elevate activity of hepatic CYP7A1, LXR*α*, and ABCG1 to promote cholesterol-to-bile acid conversion and cholesterol efflux	[114]
	C57BL/6J mice	Upregulate LDLR and CYP7A1 gene expression to facilitate the removal of cholesterol via fecal excretion	[115]
	Wistar rats	Promote conversion of cholesterol to bile acids and cholesterol efflux by increasing hepatic CYP7A1 activity and hepatic LXR*α*, ABCG1, and LDLR protein expressions	[116]
	ApoE^−/−^ mice	Elevate cholesterol accepting ability of HDL and increase ABCA1/G1 expression levels of proteins related to RCT	[117]
	Sprague-Dawley rats	Influence gene and protein expression of SREBP1c and HMGR to lower lipid	[118]
	Sprague-Dawley rats	Upregulate hepatic gene expression CYP7A1, LXR*α*, ABCA1, and ABCG1 to promote cholesterol efflux	[119]
	Zucker rats	Lower the level of HDL cholesterol and increase phosphorus level by increasing the ratio of ASAT/ALAT activity induced by leptin	[56]
	ApoE^−/−^ mice	Regulate expressions of ABCA1, LXR*α*, and PCSK9	[26]
	ApoE^−/−^ mice	Downregulate PCSK9 and CD36 protein expression and upregulate PPAR *γ*, LXR*α*, and ABCA1 protein expression levels in both the aortic and liver tissues	[65]

Alter the gut microbiota	ApoE^−/−^ mice	Regulate primary bile acid biosynthesis	[95]
	C57BL/6J mice	Improve composition and functionality of gut microbiome and production of short chain fatty acids	[120]
	Wistar rats	Stimulate bacterial enzymatic activity and increase enzymatic activity of the intestinal microbiota	[121]
	Ldlr^−/−^ mice	Reduce MDA, cholesterol, and LPC 18 : 1 and increase IL-6 and coprostanol levels	[98]

*Humans*			
Anti-inflammation	Healthy nonsmokers	Inhibit production of IL-1*β*, TNF-*α*, IL-6, and IL-8 in Lipopolysaccharide-stimulated	[122]
	CVD patients	Decrease transcriptional activity of NF-kB	[74]
Endothelial protection	CVD individuals	Enhance NO bioavailability, possibly by stimulating eNOS activity	[123]

**Table 2:** Summary of the main effects of quercetin on antiatherosclerosis in vitro.

Effect	Cell line	Target and mechanism	Reference
*Cells from animals*			
Anti-inflammation	RAW264.7 cells	Downregulate IL-1*α*, IL-1*β*, IL-2, IL-10, MCP-1, COX-2, MMP-1, SOCS3 induced by LPS and suppress LPS-induced the phosphorylation of STAT3	[18]
	RAW264.7 cells	Suppress NF-*κ*B and MAPK signaling in LPS-stimulated	[124]
	VSMCs	Suppress inflammation response via inactivating NF-*κ*B and suppressing proinflammatory gene expression	[78]
	DCs	Inhibit DC maturation via upregulation of Dabs and downregulate the Src/PI3K/Akt-NF-*κ*B-inflammatory pathways	[111]
	RAW264.7 cells	Attenuate secretion of TNF-*α* or MCP-1	[125]

Endothelium protection	RAW264.7 cells	Ameliorate overproduction of ROS induced by oxLDL	[18]
	MPMs	Inhibit ROS formation and block the vital step in activation of NADPH oxidase-membrane translocation of p47phox	[16]
	RAW264.7 cells	Protected sialic acid against H_2_O_2_-induced degradation	[125]

Lipid lowering	RAW264.7 cells	Ameliorate lipid deposition	[18]
	Raw 264.7 cells	Improve the protein expression of ABCG1 and ABCA1 to promote cholesterol efflux	[125]
	Raw 264.7 cells	Upregulate the protein expression of ABCA1, ABCG1, and LXR*α* and downregulate the protein expression of PCSK9, P53, P21, and P16	[126]

Antiapoptosis	NRK cells	Activate autophagy	[127]
	RAW264.7 cells	Promote autophagy by increasing expression of LC3-II/I and Beclin1 and block the expression of MST1 induced by ox LDL	[86]
	RAW264.7 cells	Activate the PI3K/AKT pathway	[78]
	EPCs	Activate ERK and the ERK signaling pathway	[113]

*Cells from human*			
Anti-inflammation	HUVECs	Attenuate caveolin-1 expression in endothelial cells	[128]
	HUVECs	Reduce intracellular ROS and inhibit of NF-*κ*B and AP-1 activation	[129]
	HUVECs	Downregulate mRNA expression of MCP-1 and alleviate nuclear translocation of NF-*κ*B p65 subunit through attenuating the TLR-NF-*κ*B signaling pathway	[32]
	PBMCs	Suppress cytokine and TNF-*α* release by modulating TLR-NF-*κ*B signaling pathway and TLR-mediated MAPK pathway	[77]
	HUVECs	Inhibit expression of proinflammatory factors and endothelin-1	[130]
	VSMCs	Inhibit TNF*α*-mediated vascular inflammatory responses	[131]
	HUVECs	Inhibitory effect on MCP-1 as well as inflammatory cytokines including IL-1*β*, IL-6, and TNF-*α*	[132]

Anti-apoptosis	HaVSMCs	Decrease relative expression levels of members of the mitochondrial apoptotic pathway, including P53, puma and Noxa, caspase-3, and caspase-8	[133]
	EA.hy926 cells	Regulate Akt/GSK3*β* signaling pathway by increasing the expression of p-Akt and p-GSK3*β*	[134]

Lowering lipid	Human HepG2 cell line	Promote selective uptake of HDL-C by enhancing SR-BI expression through stimulating the PPAR *γ*/LXR*α* pathway.	[61]
	THP-1 macrophages	Increase ABCA1 expression and cholesterol efflux through LXR*α* pathway to promote RCT	[62]
